# Suspected Diphtheria Toxoid and Tetanus Toxoid (dTdap) Booster Vaccine-Induced Postural Orthostatic Tachycardia Syndrome (POTS)

**DOI:** 10.7759/cureus.64008

**Published:** 2024-07-07

**Authors:** Arun R Katwaroo, Krishna Mohan Chandolu, Valmiki K Seecheran, Priya Ramcharan, Rajeev V Seecheran, Stanley Giddings, Neal Bhagwandass, Naveen A Seecheran

**Affiliations:** 1 Internal Medicine, Trinidad Institute of Medical Technology, St. Augustine, TTO; 2 Public Health, Eastern Regional Health Authority, Sangre Grande, TTO; 3 Internal Medicine, Eric Williams Medical Sciences Complex, Mt. Hope, TTO; 4 Cardiology, Eric Williams Medical Sciences Complex, Mt. Hope, TTO; 5 Internal Medicine, University of Kansas Medical Center, Wichita, USA; 6 Infectious Diseases, The University of the West Indies, St. Augustine, TTO; 7 Nephrology, The University of the West Indies, St. Augustine, TTO; 8 Cardiology, The University of the West Indies, St. Augustine, TTO

**Keywords:** adverse event following immunization (aefi), vaccine adverse event (vae), dysautonomia, autonomic dysfunction, diphtheria toxoid and tetanus toxoid (dtdap), postural orthostatic tachycardia syndrome (pots)

## Abstract

We describe a case of a 40-year-old South Asian woman who presented with symptoms suggestive of postural orthostatic tachycardia syndrome (POTS) following a diphtheria toxoid and tetanus toxoid (dTdap) booster vaccination administered one week prior. The patient’s POTS responded favorably to treatment with low-dose fludrocortisone and ivabradine. Clinicians should maintain a high index of suspicion for POTS as a possible vaccine adverse event (VAE) post-dTdap booster inoculation and be aware of appropriate management strategies.

## Introduction

*Clostridium tetani*, a gram-positive, spore-forming anaerobic bacillus, is the etiological agent of tetanus. Tetanus spores exhibit fastidious resilience and are near-ubiquitous within the environment [1]. Clinically, tetanus manifests in three primary subtypes: generalized, cephalic, and localized tetanus. Generalized tetanus affects approximately 80% of reported cases and is characterized by the classic triad of symptoms: trismus, opisthotonus, and risus sardonicus [2]. Vaccination programs, particularly those incorporating the diphtheria toxoid and tetanus toxoid (dTdap) vaccines, have significantly attenuated the global burden of tetanus [3].

According to numerous clinical reports, a range of vaccinations has been implicated with neurological conditions, such as febrile seizures, vaccine-associated paralytic poliomyelitis (VAPP), and postural orthostatic tachycardia syndrome (POTS). Despite the effectiveness of vaccination in reducing the incidence of preventable illnesses, these potential associations warrant further investigation [4]. While an exceedingly rare occurrence, some studies suggest a possible link between certain vaccinations and the development of POTS. For example, human papillomavirus (HPV) vaccination programs have demonstrably reduced HPV infection rates, but concerns regarding rare vaccine adverse events (VAEs), such as POTS, have emerged [5,6,7]. There is also mounting evidence that suggests that coronavirus-2019 (COVID) infection and its vaccination protocols can induce autonomic dysfunction, including POTS and inappropriate sinus tachycardia (IST) [8,9,10].

In light of these suspected neurocardiogenic links with vaccination, we present a unique case, a 40-year-old South Asian woman who developed symptoms suggestive of POTS one week post-dTdap booster vaccination.

## Case presentation

A 40-year-old South Asian woman with an unremarkable medical history presented to the emergency department with a one-week history of recurrent presyncope and syncope. The presyncope episodes manifested as prodromal faintness, lightheadedness, and dizziness. Syncopal events were fleeting and devoid of any ictal features. The patient reported experiencing in excess of 10 episodes of both presyncope and syncope daily. She did not report any antecedent infection or recent ill contacts upon evaluation. The patient denied head trauma, current prescription, complementary and alternative medication use, having pets, or pertinent travel history. She maintained her usual dietary intake and physical activity levels, with no lifestyle modifications preceding the onset of symptoms. She also reported being physically active before the development of symptoms and exercising regularly. Notably, the patient received a dTdap booster vaccination one week prior to the emergence of the symptom complex. Historically, she also completed a two-dose series, including a subsequent booster of the COVID-19 vaccine, approximately 15 months earlier. COVID-19 polymerase chain reaction testing performed upon presentation returned a negative result.

**Figure 1:**
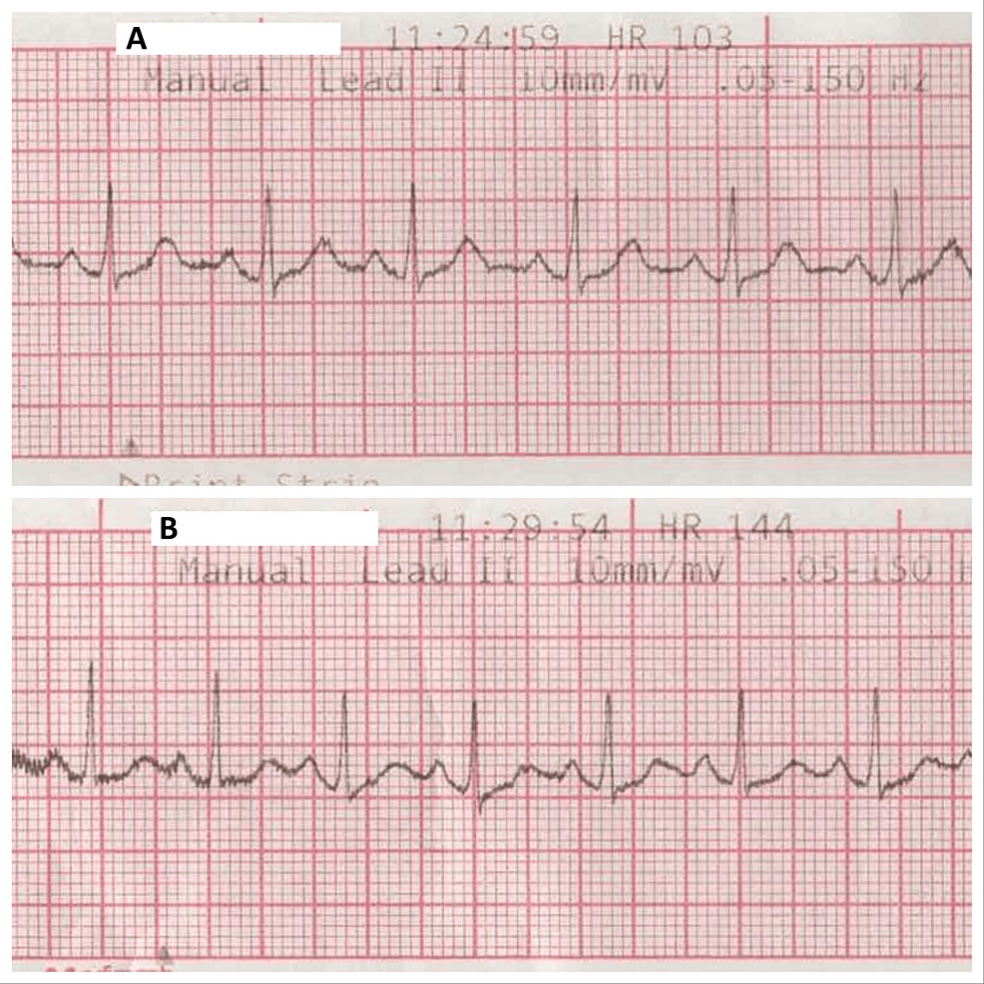
The patient's electrocardiographic tracings from tilt-table testing. A. This strip was performed at baseline and demonstrated a resting heart rate of 103 beats per minute. B. This strip was performed five minutes into tilt-table testing and revealed a greater than 30 beats per minute increase in heart rate (144 beats per minute) without any significant change in blood pressure.

**Figure 2:**
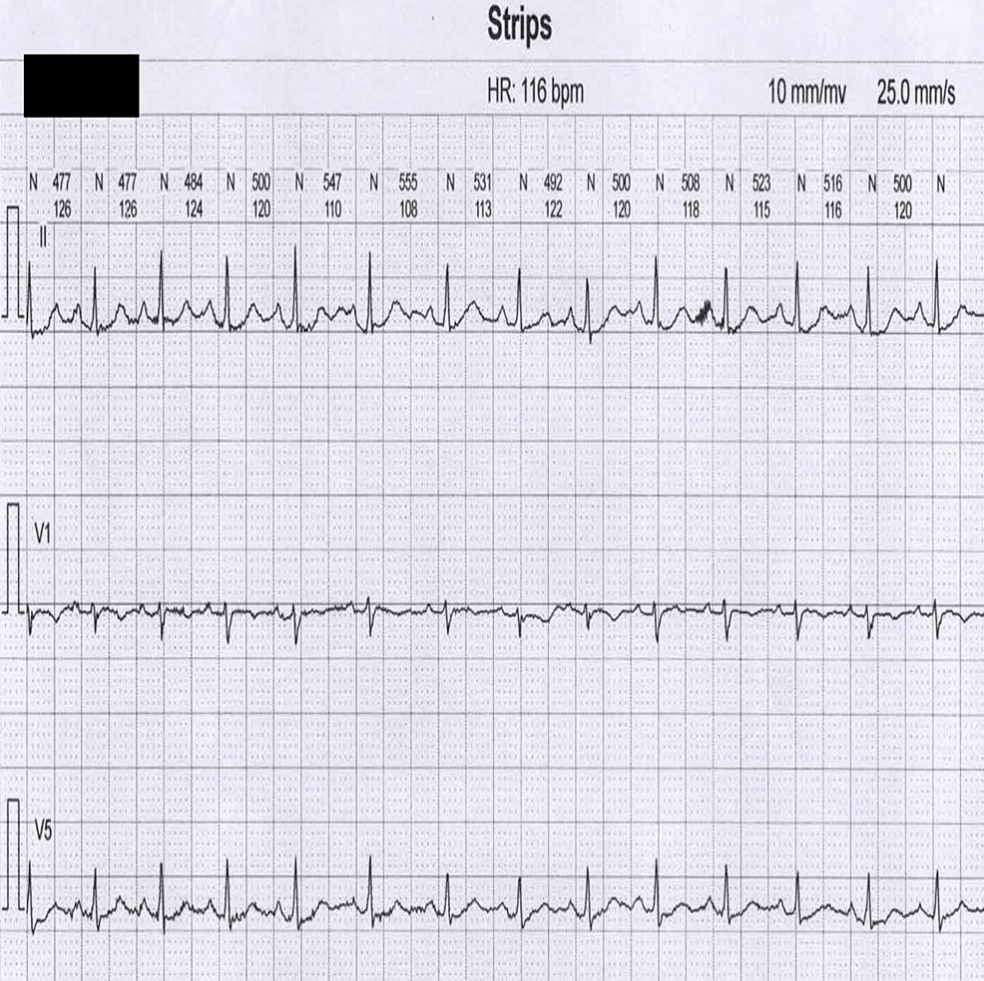
An electrocardiographic tracing from the patient's 72-hour Holter monitor.

The initial evaluation revealed the following vital signs: a blood pressure of 112/57 mmHg, a heart rate of 110 beats per minute (bpm), and oxygen saturation of 99% on room air. The patient was afebrile and had a body mass index of 26.5 kg/m². Neurological examination demonstrated an alert and oriented patient with no focal deficits. Cardiopulmonary auscultation revealed normal heart sounds and vesicular breath sounds bilaterally. Peripheral edema was absent. An extensive workup, including a two-dimensional transthoracic echocardiogram, brain computed tomography, magnetic resonance imaging with angiography, and 24-hour ambulatory electroencephalogram, were all unremarkable and did not reveal any significant abnormalities. The 72-hour Holter did demonstrate persistent sinus tachycardia and head-up tilt-table testing confirmed POTS (Table 1, Figure 1, Figure 2). Following a passive head-up tilt-table testing, the patient’s heart rate increased by >30 bpm to a maximum of 144 bpm, clinching the diagnostic criteria for POTS. The patient’s heart rate returned to baseline upon returning to a prone position. The patient did not experience significant blood pressure changes or symptoms during the tilt-table testing despite the administration of isosorbide dinitrate at a 70-degree tilt angle.

**Table 1:** The patient's tilt-table results, affirming the postural orthostatic tachycardia syndrome (POTS) diagnosis.

Time	Systolic blood pressure (SBP in millimeters of mercury, mmHg)	Diastolic blood pressure (DBP in millimeters of mercury, mmHg)	Heart rate (beats per minute, bpm)	Position
11:15 AM	111	83	103	Supine
11:20 AM	123	81	144	Head up
11:25 AM	121	89	120	Head up
11:30 AM	123	90	132	Head up
11:35 AM	131	79	117	Head up
11:40 AM	124	76	147	Isosorbide dinitrate 5 milligrams
11:42 AM	141	88	152	Head up
11:44 AM	139	87	154	Head up
11:46 AM	136	77	158	Head up
11:48 AM	131	82	160	Head up
11:50 AM	124	82	155	Head up
11:52 AM	127	79	165	Head up
11:55 AM	121	78	130	Supine
12:00 PM	122	82	128	Supine
12:05 PM	121	79	107	Supine

The patient’s three-day hospitalization course included the initiation of a pharmacotherapeutic regimen for POTS. She received fludrocortisone 0.1 mg orally every eight hours and ivabradine 2.5 mg every 12 hours. In addition, she was counseled on lifestyle modifications to optimize her circulatory status and mitigate symptoms. These recommendations included increased dietary sodium and fluid intake, sun exposure minimization to prevent dehydration, and participation in a graded moderate-intensity exercise program. The patient demonstrated a progressive clinical response, and her constellation of symptoms, including exercise intolerance and fatigue, gradually improved. The frequency of syncopal episodes subsequently decreased on consecutive days, ultimately resolving by discharge. At a one-month follow-up appointment, the patient’s cardiovascular medications were successfully de-escalated to low-dose fludrocortisone and ivabradine once daily. She reported no interval serious adverse events (SAEs).

## Discussion

Tetanus toxoid is a vaccine approved by the Food and Drug Administration (FDA) to prevent tetanus, which can be combined with other vaccines. It targets the neurotoxin tetanospasmin, which inhibits the release of a key inhibitory neurotransmitter, leading to uncontrolled muscle contractions and spasms [11]. Adverse events following immunization (AEFI) include any untoward medical occurrences post-vaccination that do not necessarily imply causation. Tetanus toxoid vaccination demonstrates a favorable safety profile, with most symptoms categorized as mild and localized and include pain, erythema, and induration at the injection site, typically resolving within 48 hours. Although less frequent, systemic reactions may encompass low-grade fever, headache, fatigue, and nausea, which are also transient [11,12]. Some life-threatening AEFIs include anaphylaxis, Guillain-Barré syndrome (GBS), and thrombosis with thrombocytopenia syndrome (TTS), which can result in protracted morbidity and be fatal [12]. A case report previously documented extreme cachexia (wasting syndrome) associated with severe dysautonomia, potentially stemming from autoimmune/inflammatory syndrome induced by adjuvants (ASIA) syndrome triggered by the Tdap vaccine [13]. However, this current discussion focuses on a case of suspected POTS following dTdap booster vaccination.

POTS is a condition characterized by orthostatic intolerance, an autonomic nervous system dysfunction affecting blood volume regulation. The main diagnostic criteria of POTS is significant orthostatic tachycardia, where the heart rate increases by at least 30 beats per minute upon standing, with minimal or no accompanying orthostatic hypotension [14,15,16,17]. Symptoms commonly associated with POTS include generalized fatigue, nausea, dizziness, heart palpitations, near syncope (presyncope), lightheadedness, and exercise intolerance. The diagnosis of POTS underscores the absence of alternative explanations for orthostatic tachycardia, such as prolonged bed rest, medications known to impair autonomic function, and chronic debilitating disorders that can induce tachycardia [14]. There is a predilection in females, most frequently occurring between 15 and 40 years of age [9,18,19]. Recent research suggests that POTS represents an accentuated response to physiological changes during orthostasis. In healthy individuals, assuming an erect position usually leads to blood volume pooling in the lower extremities due to gravity, which triggers a neural reflex that promotes peripheral vasoconstriction, enhancing venous return to the heart and maintaining blood pressure. However, in patients with POTS, this compensatory mechanism appears maladaptive and dysfunctional, where an alternative neural pathway is activated, leading to tachycardia as a means to maintain cardiac output [8,18].

Clinical evaluation of POTS commences with a comprehensive clinical assessment to identify the salient features described above. A critical differential diagnosis worth considering is pheochromocytoma, which can mimic POTS due to paroxysmal episodes of hyperadrenergic symptoms [20]. A complete blood count and electrolyte panel can also exclude anemia or electrolyte derangements. Electrocardiography or further detailed evaluation with a Holter monitor or event recorder is usually performed to assess for sinus node origin of tachycardia and to exclude the presence of accessory bypass tracts or other cardiac conduction abnormalities. Normal left ventricular function is integral in establishing the diagnosis.

Initial therapeutic strategies for POTS focus on addressing potentially reversible causes and optimizing any underlying comorbid conditions. Patient education is a cornerstone of treatment, empowering individuals to understand and manage their symptoms. In addition, POTS patients are encouraged to avoid exacerbating factors such as dehydration and extreme heat exposure [21]. The first step in pharmacological treatment is to discontinue medications that might contribute to tachycardia [22]. Beta-adrenergic blockers are frequently utilized; however, they can be counterproductive if the heart rate increase in POTS is purely compensatory. In the chronic setting, it has been demonstrated that long-acting propranolol was as effective as exercise in lowering standing heart rate but did not enhance the quality of life for POTS patients [23]. In addition, it has been shown that a non-selective beta-blocker may be more effective than a selective beta-blocker since it also blocks beta-2 adrenoreceptor-mediated vasodilation [22,24]. Fludrocortisone, an analog of aldosterone, is commonly prescribed for patients with known or strongly suspected hypovolemia, attributed to increasing plasma volume by promoting sodium retention. However, potential adverse effects may include significant hypokalemia, headaches, acne, and fluid retention, leading to edema [22]. Ivabradine exerts its effect by prolonging diastole and reducing heart rate. This action is achieved through selective inhibition of the If (funny) current channels responsible for the cardiac pacemaker current. These transmembrane ion channels conduct an inward depolarizing current composed of sodium and potassium ions. By selectively blocking these channels, ivabradine slows heart rate without affecting systemic vascular resistance or cardiac inotropy [25,26]. While not currently FDA-approved for POTS, ivabradine’s ability to reduce heart rate has shown fair promise in managing symptoms [26,27,28,29].

This case presentation highlights a possible association between dTdap booster vaccination and the subsequent development of POTS in a 40-year-old female patient. The temporal relationship between vaccination and symptom onset, confirmed diagnosis via tilt table testing, absence of alternative etiologies, and concordance with typical POTS demographics strengthen the argument for a vaccine-adverse event (VAE). Reporting this case to the Vaccine Adverse Event Reporting System (VAERS) contributes to ongoing vaccine safety surveillance.

## Conclusions

In this report, we presented a case of a 40-year-old South Asian woman who developed symptoms consistent with POTS one week following a dTdap booster vaccination. The patient’s clinical presentation and diagnostic workup confirmed POTS, which responded favorably to a treatment regimen of fludrocortisone and ivabradine. This case presentation contributes to the emerging literature exploring AEFI and VAEs associated with dTdap vaccination.
